# Growth-restricting effects of siRNA transfections: a largely deterministic combination of off-target binding and hybridization-independent competition

**DOI:** 10.1093/nar/gky798

**Published:** 2018-09-12

**Authors:** Neha Daga, Simone Eicher, Abhilash Kannan, Alain Casanova, Shyan H Low, Saskia Kreibich, Daniel Andritschke, Mario Emmenlauer, Jeremy L Jenkins, Wolf-Dietrich Hardt, Urs F Greber, Christoph Dehio, Christian von Mering

**Affiliations:** 1 Institute of Molecular Life Sciences, University of Zurich, CH-8057 Zurich, Switzerland; 2 Swiss Institute of Bioinformatics, University of Zurich, CH-8057 Zurich, Switzerland; 3 Biozentrum, University of Basel, CH-4056 Basel, Switzerland; 4 Institute of Microbiology, Department of Biology, ETH Zurich, CH-8093 Zurich, Switzerland; 5 Chemical Biology and Therapeutics, Novartis Institutes for BioMedical Research, 181 Massachusetts Avenue, Cambridge, MA 02139, USA

## Abstract

Perturbation of gene expression by means of synthetic small interfering RNAs (siRNAs) is a powerful way to uncover gene function. However, siRNA technology suffers from sequence-specific off-target effects and from limitations in knock-down efficiency. In this study, we assess a further problem: unintended effects of siRNA transfections on cellular fitness/proliferation. We show that the nucleotide compositions of siRNAs at specific positions have reproducible growth-restricting effects on mammalian cells in culture. This is likely distinct from hybridization-dependent off-target effects, since each nucleotide residue is seen to be acting independently and additively. The effect is robust and reproducible across different siRNA libraries and also across various cell lines, including human and mouse cells. Analyzing the growth inhibition patterns in correlation to the nucleotide sequence of the siRNAs allowed us to build a predictor that can estimate growth-restricting effects for any arbitrary siRNA sequence. Competition experiments with co-transfected siRNAs further suggest that the growth-restricting effects might be linked to an oversaturation of the cellular miRNA machinery, thus disrupting endogenous miRNA functions at large. We caution that competition between siRNA molecules could complicate the interpretation of double-knockdown or epistasis experiments, and potential interactions with endogenous miRNAs can be a factor when assaying cell growth or viability phenotypes.

## INTRODUCTION

Despite immense progress in gene editing technologies such as CRISPR/Cas9 (1,2), transient siRNA transfection remains a powerful technique for perturbing gene function in the lab. The siRNAs (*short interfering RNAs*) bind target mRNAs via sequence complementarity, and interfere post-transcriptionally with gene expression by inducing mRNA degradation and/or by blocking translation (3–5). The siRNA technique has advantages particularly in high-content settings, where phenotypes can be analyzed in high-throughput manner using automated microscopy at sub-cellular resolution level (5,6) or in settings where a complete removal of a gene's function may be undesirable due to lethality. siRNA interference has yielded critical insights into the genetic foundations of a large number of phenotypes, from cancer (7–12) to development (13–15) to pathogen infections (16–21).

To achieve delivery of siRNAs into the cell, a number of approaches have been established, including expression from within the genome as short hair-pin precursor molecules (shRNA), or transfection of synthetic mixtures of double-stranded RNA molecules derived from longer precursors (esiRNA). However, the most widely used delivery approach relies on commercially available libraries consisting of defined, double-stranded siRNA oligonucleotides (oligos). These oligos are designed to uniquely bind to a single target mRNA transcript, are transfected individually or in pools, and are available at a genome-wide scope.

Perhaps owing to its maturity and wide-spread adoption, a number of critical issues with the siRNA technology have been identified. First, the knock-down efficiency that can be achieved is typically not 100%, and can vary from gene to gene and from siRNA to siRNA. Secondly, many siRNA oligos will bind not only to the intended target transcript, but in addition also to a number of other, unrelated transcripts via partial sequence complementarity. This leads to so called ‘off-target’ effects, and is thought to be a consequence of siRNA oligos entering the endogenous miRNA pathway, where they function essentially as miRNAs with a concomitant broader range of targets. Off-target effects are highly reproducible, and their specificity is mostly determined by a short stretch of nucleotides within the siRNA molecules (nucleotide positions 2–8, corresponding to the ‘seed’ region in normal miRNAs) (22–24). Depending on the phenotypic consequences, seed-based off-target effects can dominate over the intended on-target readouts, and may limit the discovery power of any given screen (24,25).

In addition, practitioners of siRNA screening will often observe that the siRNA transfections *per se* affect cellular fitness. At the time points that are typically chosen for phenotypic readouts (i.e., usually a few days after transfection) the number of cells in transfected populations is often noticeably smaller than that in mock-transfected populations, even for non-targeting, ‘negative-control’ oligos. The underlying mechanisms are still not entirely resolved. RNAi mediated reductions in cellular fitness/proliferation have been hypothesized to be due either to innate immunity mechanisms responding to the presence of exogenous RNA molecules (26–28), or to competitive interactions of the siRNA molecules with endogenous RNAs, for example with miRNAs that might be displaced from the endogenous miRNA machinery (29–31).

For the present study, we have carefully analyzed determinants of cellular fitness/proliferation in relation to the oligonucleotide sequences, across a number of screens conducted by different laboratories using different commercial siRNA libraries. We uncovered consistent fitness/proliferation effects of transfected siRNA oligos, independent of their intended on-target activity. We find that in part, these effects can be attributed to previously described, seed-dependent off-target effects. However, we find an additional, novel determinant, which is not a function of extended nucleotide sequence context as would be expected for any hybridization-dependent mechanism. Instead, individual nucleotides within the oligos have independent, additive and position-specific effects on cellular growth/survival, and this can be modeled with simple linear regression. We hypothesize that this observation might be due to competition between external and endogenous RNA molecules for some endogenous cellular binding partner. We can indeed reproduce such a competition among oligos in specific co-titration experiments. The strength of this competition appears to be mostly a function of local sequence composition, allowing us to create a generalized software predictor for ‘cellular fitness/proliferation’ consequences upon transfection of any given siRNA oligo sequence.

## MATERIALS AND METHODS

### Data sets

The present study is based on a number of large-scale siRNA screens, which were originally carried out in the context of a comparative project on host-pathogen interactions (24,32). If not stated otherwise, here we are concerned not with their infection readouts, but with the readouts in terms of the number of cells after each perturbation. The presence of the pathogens did not influence the cell numbers in a systematic way (data not shown), and our observations were reproducible across all the distinct pathogens studied (four viruses, five bacteria). The image processing and data normalization of the screens have been described previously (24,32). The final cell-number phenotypes were expressed as *z*-scores relative to the screening background of each dataset. All processed and normalized, oligo-resolved phenotypes are available as Supplementary Data Files accompanying the manuscript, together with siRNA sequence information (to the extent permitted by the commercial library providers).

### Cell number predictors

The siRNA nucleotide compositional profiles seen in Figure 2 were obtained by aggregating, for each datapoint, all those siRNAs that happened to have a specific nucleotide at one specific position (typically resulting in several thousand oligos per datapoint). This was carried out for each of the four nucleotides and over the entire range of possible positions of siRNAs, and the average phenotype of each group was plotted. These positional profiles were then compared to each other for different cell lines, vendors, organisms, and phenotypes analyzed here, and their distances were visualized in a PCoA plot.

A predictor for relative cell number, given an arbitrary siRNA sequence, was constructed separately for each organism and library type. The predictor uses a multiple linear regression model, with learned weights for each position. Separately, a model was trained based on seed sequences only—for this, not the individual nucleotide positions, but the entire 7-mer sequences covering oligo-positions 2–8 constituted the data points. Both predictors were then combined in a weighted linear fashion; the best performance was seen at 50% relative weight for both (Figure 4).

### Model validation using custom-designed siRNAs

Specific siRNA oligos were designed such that each had a previously unobserved sequence, and no intended on-target gene in the genome. Their nucleotide sequences were arbitrarily specified, sampling for each position a nucleotide according to the position-specific weights of the linear regression model, aiming to design oligos of three classes: either to reduce cell counts (‘growth-restricting’), to leave cell counts unaffected (‘non-growth-restricting’), or to be representing the background frequencies (‘randomly sampled’) of nucleotides in the entire siRNA library (i.e. randomly sampled controls). All of these designed customized sequences were ordered from Qiagen using their HP custom siRNA synthesis option.

Human cervix carcinoma HeLa CCL2 cells (ATCC), human airway epithelial A549 cells, and human diploid WI38 fibroblasts were grown in Dulbecco Modified Eagle Medium (DMEM, Sigma) supplemented with 10% heat inactivated Fetal Calf Serum (FCS— Invitrogen) at 37°C and 5% CO_2_ and 95% humidity. All transfection experiments were carried out in a 384-well plate format using a protocol for reverse transfection, cell fixation, staining, and imaging as described in (32). Cell numbers used here were 700 cells/well (A549, HeLa CCL2 ATCC) or 1000 cells/well (WI38). Cells were stained with DAPI and their microscopy images were analysed with Cell Profiler (33) using a custom-made pipeline allowing the detection of nuclei. Nucleus objects labelled ‘Nuclei’ were segmented in the DAPI channel using OTSU’s method (Cell Profiler module IdentifyPrimAutomatic), and counted to establish reliable cell counts.

### Oligo-oligo competition assays

HeLa CCL2 cells were maintained in Dulbecco's modified Eagle's medium (DMEM, Sigma) supplemented with 10% heat inactivated fetal bovine serum (FBS, Gibco). siRNAs were spotted in a random array into 384-well μClear Plates (Greiner). Wells were loaded with 1.6 pmol of customized siRNAs and with variable amounts of siRNA targeting the KIF11 transcript (2-fold dilution series: range 9.77 × 10^−5^–25.6 pmol) in a final volume of 5 μl of RNAse-free ddH_2_O (Ambion).

For transfection, spotted siRNAs were complexed in 25 μl of DMEM supplemented with 1:250 Lipofectamine RNAiMAX (Invitrogen) at room temperature for 1 h. Subsequently, 600 cells/well were seeded in 50 μl of DMEM/16% FCS to result in 10% FCS concentration. Final siRNA concentrations were 20 nM for custom siRNAs combined with diverse concentrations of siRNA targeting KIF11 (1.95 × 10^−5^ μM up to 5.12 μM) as indicated in Figure 5. Cells were incubated at 37°C and 5% CO_2_ for 72 h. Cells were then fixed in 3.7% PFA/HEPES (Sigma, PanReac AppliChem) for 10 min and were permeabilized by 0.1% Triton X-100 (Sigma) in PBS for 10 min. DNA was stained by 1 μg/ml DAPI (Roche) in PBS. Imaging and analysis: per condition nine images were acquired at an ImageXpress system (Molecular Devices) with a 10× S Fluor objective (Nikon). Images were analysed with the screeningBee analysis framework (https://www.screeningbee.org). To correct for uneven illumination from widefield microscopy imaging, the model for CIDRE illumination correction was computed from all images (34). To ensure that CIDRE-corrected image intensities fall within the [0.0, 1.0] range, a linear intensity transformation for pixel intensities was computed that maps the 0.001-quantile to 0.01 and the 0.999-quantile to 0.99 after illumination correction. On illumination-corrected DAPI images, Cell Profiler 1 (33) was executed to perform object segmentation and measurements: nuclei were identified using ‘OTSU Global’ segmentation. The median OTSU value was manually set as segmentation threshold for the images.

Data from the co-transfection competition experiment as seen in Figure 5 was analysed using the R-package *drc* (35). The data was subjected to non-linear regression analysis and fitted using the four-parameter log-logistic equation shown below to determine at what concentration the siRNA targeting the KIF11 transcript caused the reduction in cell count by 50%, represented by the parameter *e* of the equation.

\begin{equation*}f\left( {x,\left( {b,c,d,e} \right)} \right) = c + \frac{{d - c}}{{1 + {e^{b\;\log \frac{x}{e}}}}}\end{equation*}

The four parameters in the equation are, *c* and *d*: coefficients corresponding to the lower and upper asymptotes, *b*: the slope of the line, and *e*: the response at the inflection point halfway between the upper and lower asymptotes (also known as ED50). The inflection points computed above for siRNAs targeting KIF11 transcript with or without the presence of several customized siRNAs were subjected to ANOVA to determine whether there were any significant differences between them. This was then followed by Tukey HSD analysis to determine the pairwise significant difference between the inflection points at α = 0.05 in different scenarios as seen in Figure 5.

### AGO2 rescue assays

Analogous to the oligo–oligo competition assays, 1.6 pmol of customized siRNAs loaded per 384-well-slot were complexed. HeLa CCL-2 cells with a prior exposure of 48 h to the following treatments (siAGO2, siGFP Duplex III, untreated) were seeded onto these complexed oligos to result in a final custom oligo concentration of 20 nM per well. Cells were incubated for 72 h and thereafter processed and analysed for cell numbers. Ago2 knockdown quality after single knockdown (Supplementary Figure S2A) and consecutive double knockdowns (Supplementary Figure S2B) was assessed on Western blot (AGO2: Novus Biologicals H00027161-M01, GAPDH: EMD Millipore ABS16).

### RT-qPCR measurements to quantify interferon response genes

To measure the levels of Human MX1, IFIT1, ISG15 and OSA1, HeLa CCL-2 cells were reverse-transfected in 6-well plates with 20 nM siRNAs using 0.1% (v/v) Lipofectamine RNAiMAX (Invitrogen), according to the manufacturer's instructions. Transfection reactions were performed in serum-free OptiMEM (Gibco). Total RNA from the siRNA transfected cells was extracted using mirVana RNA isolation kit (Ambion). Following on-column DNase digestion (Qiagen), 300 ng of total RNA was reverse-transcribed using the ‘Transcriptor’ First Strand cDNA Synthesis Kit (Roche) with anchored-oligo(dT)_18_ primers. RT-qPCR was performed using Power SYBR Green Master mix (Applied Biosystems). Reactions were analyzed by an ABI 7000 real-time PCR machine using the following cycle conditions: 50°C for 10 min, 95°C for 10 min, followed by 40 cycles at 95°C for 15 s and 60°C for 1 minute. Relative mRNA levels (2^−ΔCt^) were determined by comparing the PCR quantification cycle (Cq, determined with the Software SDS 2.2.1) and normalized against the reference gene expression TATA-Box Binding Protein (TBP), elongation factor 1 alpha (EEF1A1) and transferrin receptor protein 1 (TFRC).

## RESULTS

### Non-specific effects of siRNA transfections

We analyzed raw data from several RNAi pathogen-entry screens that had been based on commercially available, de-convoluted siRNA libraries from different vendors, and had been executed in HeLa CCL2 cells (24). Three of the screens were genome-wide pathogen screens, carried out for two bacterial species (*Brucella abortus* and *Salmonella typhimurium*, see Supplement) and a virus (*Uukuniemi* virus (36)). In addition, several screens with a more restricted set of perturbed genes were also analyzed, which addressed the ‘kinome’ only, i.e. the complement of human kinases and associated proteins (32). These latter screens covered a larger number of pathogens, and used a larger number of different siRNAs per gene (from three different vendors). All screens above had been scored for infection-phenotypes as well as cell-number phenotypes. All post-processing, image analysis and normalization was standardized, with final phenotypes reported as z-scores relative to the entire screening background (32).

**Figure 1.**
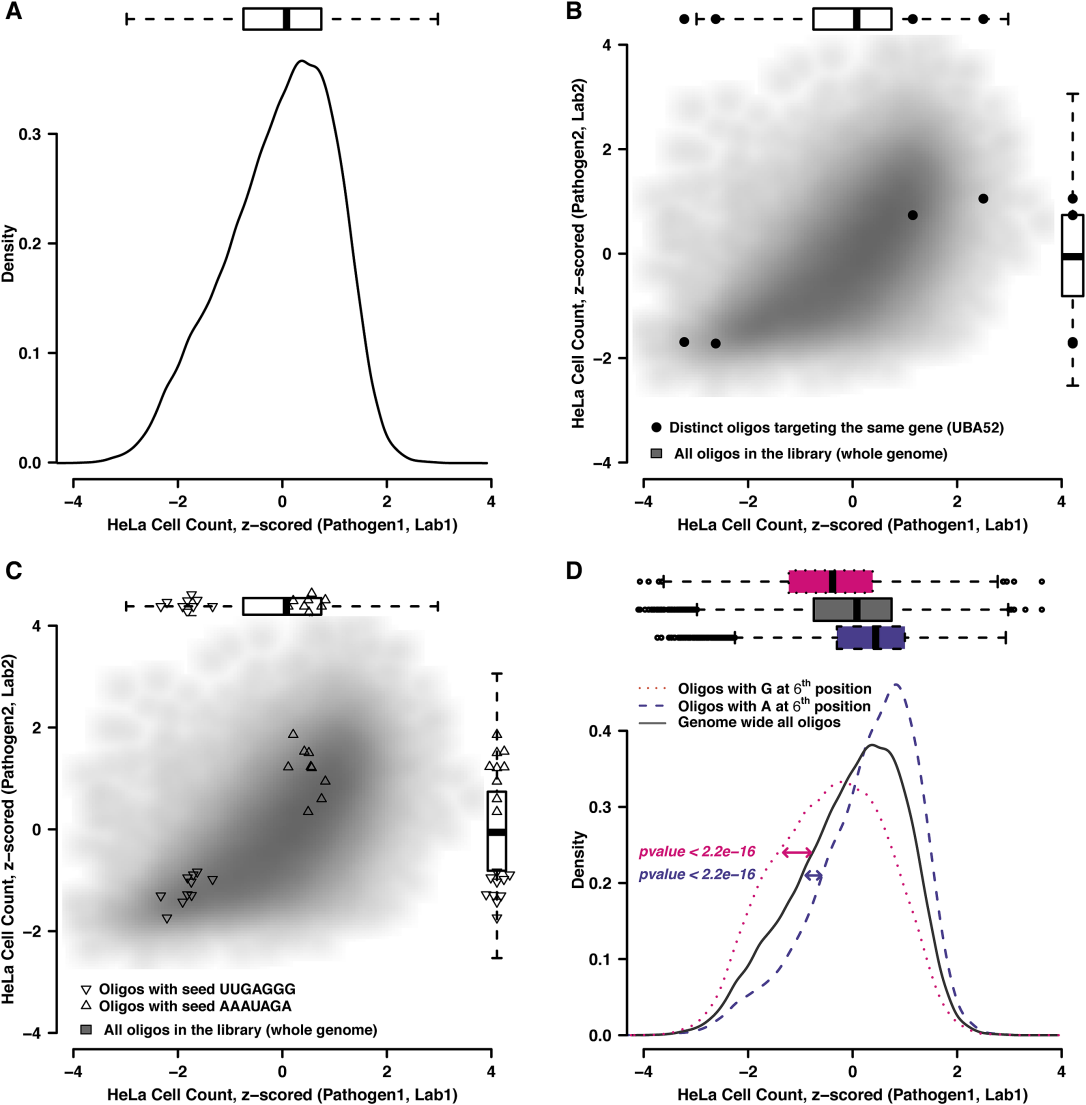
Different types of off-target effects of siRNAs in RNAi pathogen screens. (**A**) Cell number distribution from a genome-wide pathogen screen (here, *Brucella abortus*). (**B**) On-target reproducibility of the cell-number phenotype. The four data points highlighted in black represent four distinct siRNAs targeting the same gene, shown against the genome-wide reproducibility of identical oligos in distinct screens (gray scatter plot of 69,000 siRNAs targeting the human genome, in two pathogen screens; Pathogen1: *Brucella abortus* and Pathogen 2: *Uukuniemi virus)*. (**C**) Strong and consistent seed-mediated off-target effects of siRNAs across RNAi screens. The two clusters represent two different seed sequences, one increasing the cell number and the other decreasing it. All data points in each cluster represent oligos targeting different genes but containing the respective seed sequence of the cluster. Background scatter as in (B). (**D**) Shifted cell-count distributions, caused by the presence of a single nucleotide at a specified position. Three different cell count distributions are represented by three colored line types. The black solid line represents the cell count of all the siRNAs in a genome-wide screen (*Brucella abortus* screen), pink dotted and violet dashed lines represent cell count distributions of all those siRNAs in that same screen that have either adenine at 6th position or guanine at 6th position, respectively. In all panels, the box-plots denote the percentiles 25%, 50% and 75%, respectively, with their whiskers extending to the highest or lowest data points that are at most 1.5 times the box distance away from the box.

The cell-number phenotype from a typical genome-wide pathogen screen (*Brucella abortus)* follows an approximately normal distribution, with a slight excess of low-cell-count phenotypes (Figure 1A). While there is good technical reproducibility of the cell-count phenotype of a given siRNA oligo, there is a relatively poor phenotypic reproducibility across distinct siRNAs targeting the same gene, indicating a relative lack of on-target signal across all pathogens and all screens that were analyzed. This is exemplified in Figure 1B where four data points, representing four distinct siRNAs targeting the same gene, individually produce reproducible phenotypes across two independent screens but fail to agree on the actual phenotype of the gene perturbation. Globally, this behavior is observed for all phenotypes and screens tested (24) (for Adenovirus: data not shown).

siRNAs are known to be associated with different types of off-target effects (37). One of them is widely known, and occurs when siRNAs act like microRNAs by partially hybridizing through their ‘seed region’ with multiple mRNAs and thus affecting many off-target transcripts in addition to their intended targets. This seed-mediated off-target effect also dominates the cell-count phenotype in all of our analyzed screens. siRNAs sharing the same seed sequence (i.e. positions 2 to 8 of the siRNA sequence) produce similar phenotypes and cluster together. This can be seen from two exemplary seed clusters in Figure 1C, where one seed group decreases the cell number and the other enhances it. The siRNAs belonging to each of these clusters share no significant sequence similarity outside the shared seed sequence. These seed-mediated off-target effects for cell numbers are highly reproducible across the screens, even when screens were carried out independently in two different laboratories and across different pathogens (24).

However, in our study we observed an additional source of off-target phenotypes, namely that individual nucleotide positions in the siRNA molecules can have independent and additive effects on the observed cell number in all genome- and kinome-wide screens that we have analyzed. This has not been described before, to the best of our knowledge, and seems distinct from a seed-mediated off-target effect as discussed below.

The effect becomes statistically visible when analyzing an entire screen: we observed that the average cell number phenotype within a genome-wide screen (∼69 000 siRNAs targeting ∼18 000 genes) can be shifted significantly depending on the nucleotide present at a single residue position in the siRNAs, irrespective of the remainder of the sequence and irrespective of their intended targets (Figure 1D). For example, all those siRNAs from the genome-wide screen that happened to have an adenine at their 6th position together produce a shifted cell-count phenotype distribution, i.e. shifted towards higher cell numbers, as compared to, e.g. a guanine at 6th position, which shifts to a lower cell number. This was detectable for nearly all of the possible positions within siRNAs, but the magnitude and directionality of the shift depended on the type of nucleotide and its precise position within the siRNA.

### Robust positional effect of nucleotide composition on cell count

**Figure 2.**
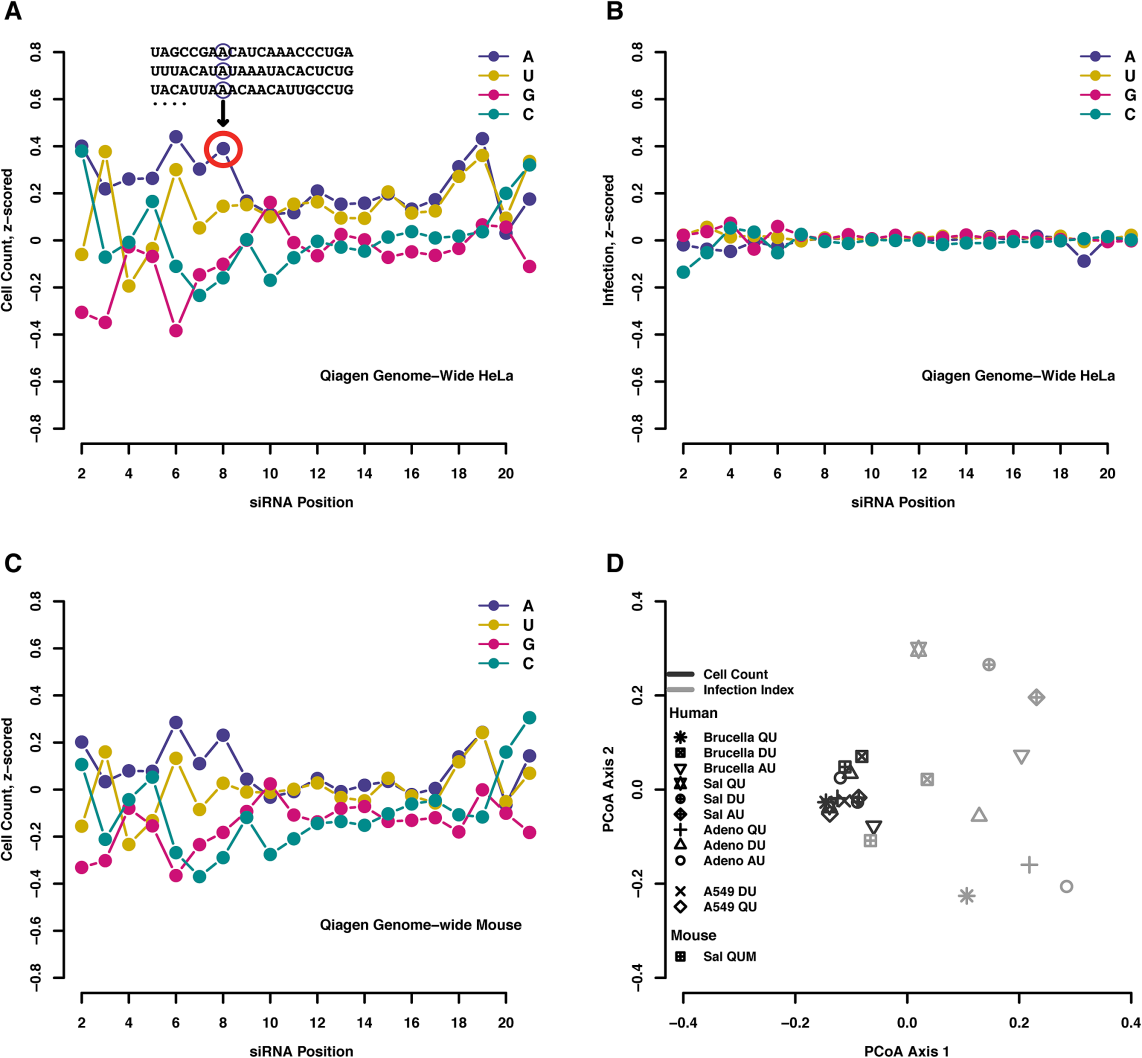
Positional profiles of phenotypic consequences of individual nucleotides at individual siRNA positions. (**A**–**C**) Each data point represents the average phenotype of all siRNAs that happen to have the specified nucleotide at that particular position. Note that the first position on the 5′-end is not considered here, since it has a strong technical bias in library design and not all nucleotides are sufficiently covered there (see also Supplementary Figure S2). (A, B) Positional profile for cell count and infection phenotypes, respectively, in a genome-wide *Brucella abortus* screen in HeLa CCL2 cells conducted with a non-pooled siRNA library (Qiagen). (C) siRNA nucleotide positional profile for cell count in a genome-wide screen for *Salmonella* entry into MEFs (murine embryonic fibroblasts, unpublished). (**D**) Global comparison of siRNA nucleotide profiles, generated for three different pathogen screens (*Brucella abortus, Salmonella* Typhimurium and *Adenovirus*), across three different libraries of non-pooled siRNAs designed by Qiagen, Dharmacon and Ambion (QU, DU and AU, respectively) targeting human kinome genes (∼ 800) in HeLa CCL2 cells, together with another genome-wide screen carried out in human A549 cells and mouse embryonic fibroblast cells using non-pooled Dharmacon (DU) and Qiagen libraries (QUM), respectively. Each data point represents the entire nucleotide profile as seen in panels A–C. Black color denotes cell count readouts and gray color denotes infection readouts.

Investigating more into the bias caused by individual nucleotides at specific position of siRNAs on the cell number phenotype, a nucleotide positional profile of all the phenotypic consequences of individual residue choices in an average siRNA emerges (Figure 2A and B). This profile was obtained by aggregating all siRNAs with a specific nucleotide at a given position into one data point (the aggregation was carried out for each of the four nucleotides and over the entire range of possible positions on siRNAs).

Remarkably, the effect is seen only in the cell-number phenotype, but not in the infection (cell-entry) phenotypes: The average cell number plotted against the positional siRNA nucleotide composition shows distinct, reproducible features (Figure 2A), whereas the same position profile applied to the infection phenotype (Figure 2B) remains essentially flat for all pathogen tested. This is of note for the general infection screening setup as well, as it confirms that the baseline infection efficiency is largely independent of the cell numbers reached in the screens (this had been controlled for in preparative cell titration experiments during assay development).

For the cell number phenotype, we observed that guanine nucleotides generally led to lower cell-numbers, but the effect is position-dependent and has reproducible exceptions at certain positions (for example position 10 where guanine residues actually resulted in *increased* cell numbers instead (Figure 2A). Also, the amplitude of the effect is not uniform across the length of the siRNA molecule, with positions closer to the 5′-end of the active guide strand generally having a stronger effect. We constructed similar profiles for each screen analyzed, and confirmed them in separate experiments in human lung epithelial A549 cells (data not shown) as well as in murine primary embryonic fibroblasts (Figure 2C). We observed that the profile is highly robust and reproducible not only across different pathogen screens and laboratories, but also across different cell lines and siRNA library types. This is evidenced by the clustering of black data points in the summary panel 2D, each representing the siRNA nucleotide positional profile of the cell number phenotype in a single screen. In contrast, the infection phenotype never resulted in a reproducible pattern, and the various screens were not clustered (light gray dots in panel 2D).

Commercial siRNA libraries are not utilizing each nucleotide at the same frequency; instead, they follow certain general design principles (38–40), although the detailed design algorithms are typically kept proprietary. We compared our observed position-dependent nucleotide profiles against plots showing these inherent ‘design-biases’ for each library (Supplementary Figure S3). We observe that the positional nucleotide biases in the three libraries are distinct from our phenotypic profiles described in Figure 2—the design biases affect different nucleotides at different positions (most strongly positions #1, #10 and #19), and the biases are not preferentially clustered towards the seed region. When including the library design bias profiles in a direct comparison with our phenotypic profiles, they clustered with each other but clearly distant from the phenotype profiles (PCoA plot in Supplementary Figure S2C, red dots). Design biases are therefore unlikely to explain the phenotypic profiles. Furthermore, it should be noted that our phenotype profiles were determined from subsets of siRNA oligos where a given position of interest was ‘fixed’ for a given nucleotide of interest (Figure 2A). With the exception of position #1, which has the strongest design bias and is not considered here, this means that several thousand oligos were available for each nucleotide of interest at each position, allowing a robust statistical inference.

### Effective cell count prediction from a model based on siRNA positional phenotype profiles

**Figure 3.**
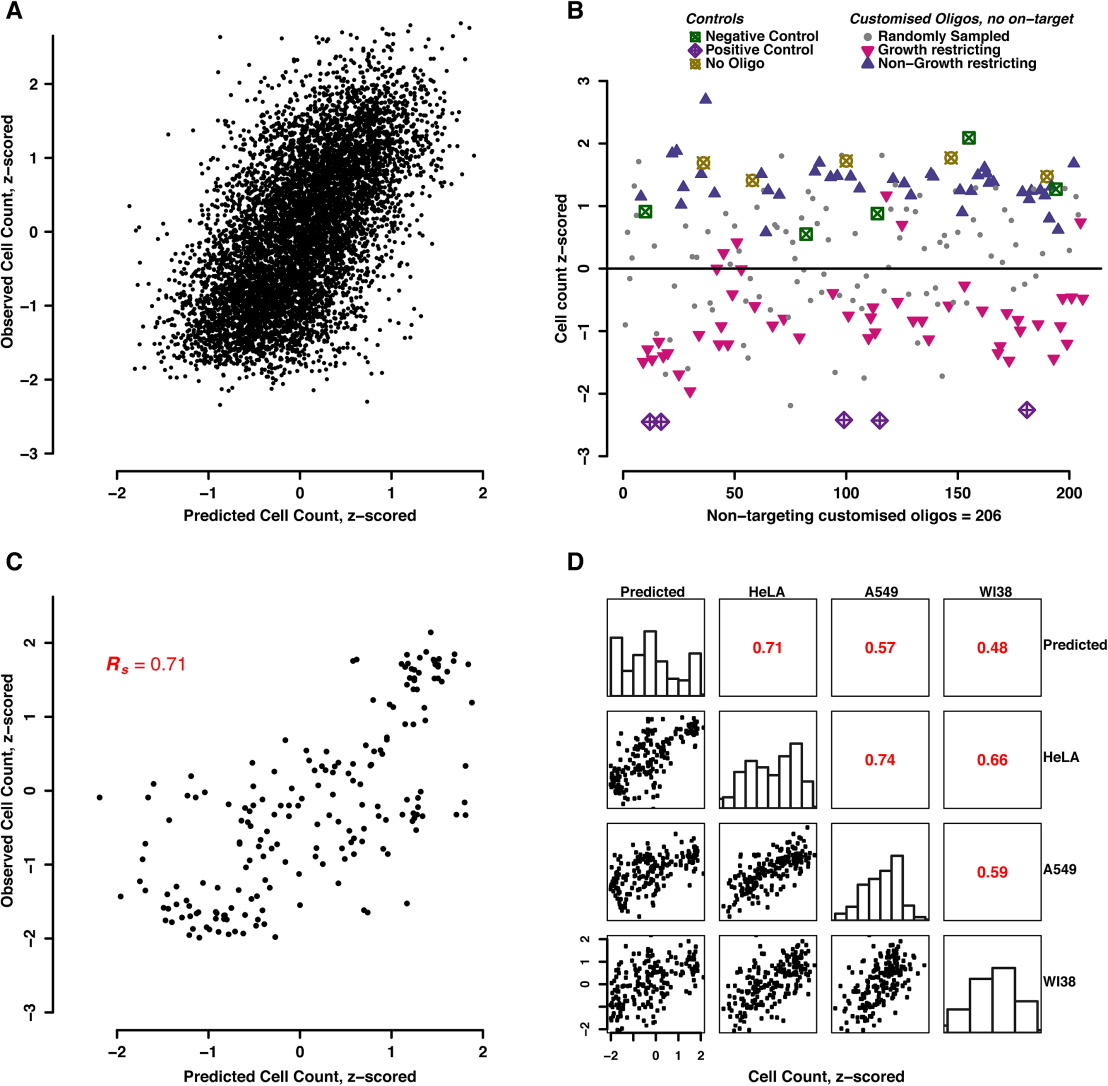
Predicting cell-number reductions from siRNA sequence. (**A**) A linear model was trained on 90% of the *Uukuniemi* genome-wide screening data and used to predict the remaining 10% of data. Correlation plot between observed and predicted values for cell count upon transfection. (**B**) Experimental design for testing the predictor with previously unobserved, arbitrary siRNA sequences. Oligos are separated by design-class, and observed phenotypes are shown. (**C**) Correlation plot between observed and predicted values of customized siRNAs in HeLa CCL2 cells. (**D**) Experimentally validating the model prediction across cell lines. The linear model trained on a genome-wide screen in HeLa CCL2 cells (*Brucella abortus* screen) was used to predict the cell counts across different cell lines. Summary/correlation plots between the observed and predicted values of customized siRNAs across cell lines. The small histograms in the center describe the distribution of data points along the x-axis, per column.

The reproducibility and robustness of the positional phenotype profile for cell counts made it seem possible to use it for predicting the cell number computationally, given only the siRNA sequence information. We set up a simple linear regression model, using 90% of the screening data for training and the rest (10%) for testing. This was done for three genome-wide pathogen screens; using the model we were able to predict the cell counts to around 55–60% accuracy in each of these screens (Figure 3A). In the next step, we validated the model by experimentally testing custom-ordered siRNA oligos with an arbitrary sequence, designed on the basis of the profile (Figure 3B). These siRNAs were non-targeting and not seen before in any of the libraries used in the studies. We designed three kinds of customized siRNAs according to the above model prediction. One class was predicted to restrict the cell number, labelled as ‘growth restricting’, the other was predicted to have minimal effect on the cell number, labelled as ‘non-growth restricting’, and the third class of ‘randomly sampled’ siRNAs was based on the library-wide background frequencies of nucleotides at each position. These oligos, together with appropriate controls, were assembled in a 384-well plate format, and tested in three human cell lines: HeLa, A549 and WI38 cells. Indeed, our model successfully predicted the transfection outcomes in terms of cell numbers, for extreme as well as for intermediate phenotypes, suggesting that it can be used to estimate phenotypes for oligos that have not been tested before.

Secondly, despite being trained on HeLa CCL2 cells only, the model was able to predict cell counts not only in HeLa cells (with up to 70% accuracy, Figure 3C) but also in A549 (cancerous cells; adenocarcinomic human alveolar basal epithelial cells) and WI38 (normal diploid fibroblasts derived from lung tissue) cells to about 60% and 50% accuracy, respectively (Figure 3D). This would hint at a common mechanism behind this growth restricting effect in all these three (different) cell lines.

### Combined cell-count predictor using both seed-based and nucleotide-based modeling

Next, our aim was to increase the power of our model by combining it with the known ‘seed-based’ off-target effects that also impact on cellular fitness/proliferation. Hence, the second part of the model was based on learning the average seed phenotypes for each 7-mer seed sequence from the screening data, unlike the first part, which was based on independent positional contribution of each of the four nucleotides at each possible position.

**Figure 4.**
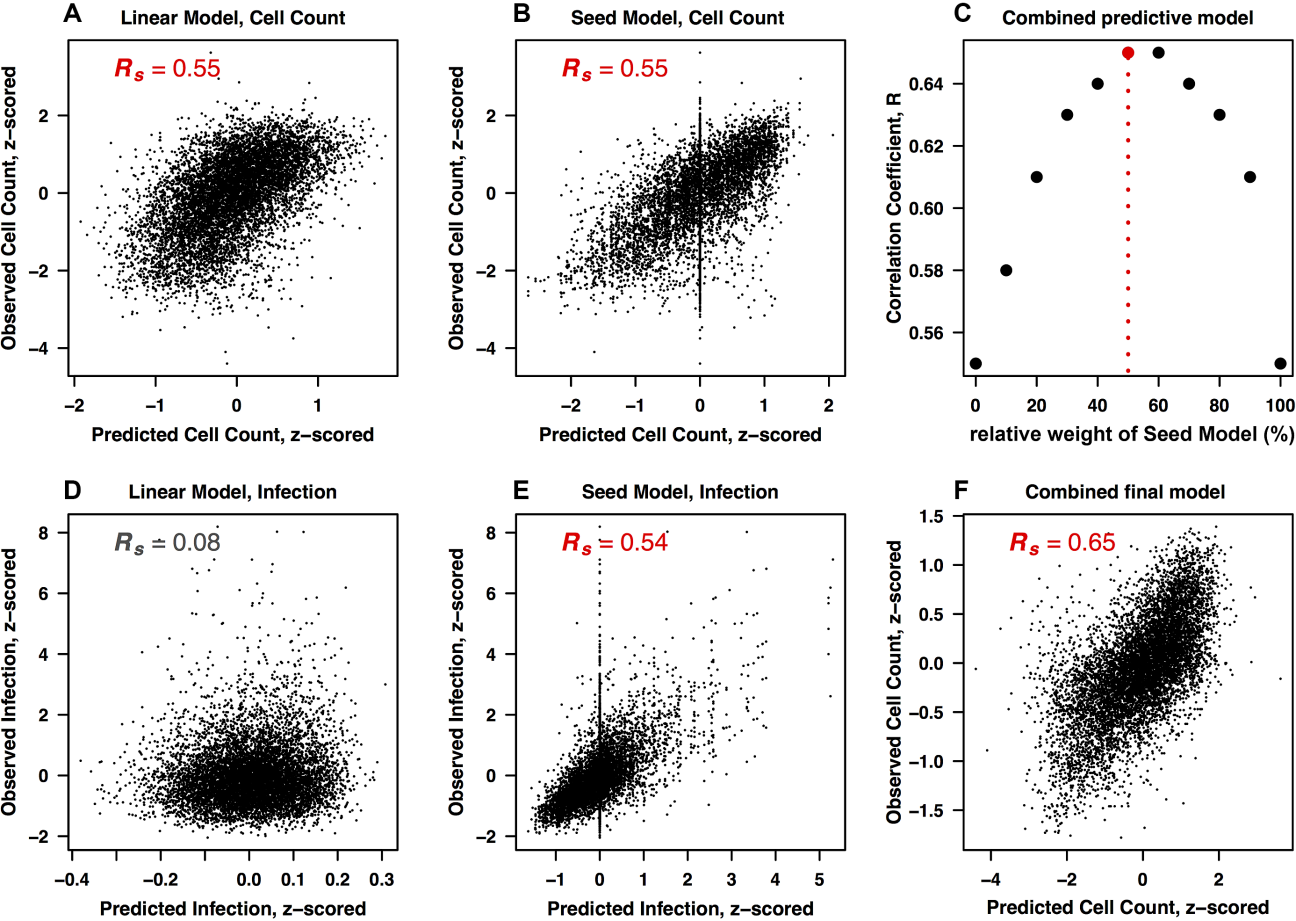
Combined modeling to increase the predictive power. (**A, B**) Performance of the two individual models for predicting the cell growth phenotype, trained (A) on individual nucleotide positions, and (B) on 7-mer seed sequences at position 2–8. (**D, E**). Corresponding plots for the infection phenotype. Plots show correlations between the observed and predicted phenotypes. (**C**) Combining seed model and linear nucleotide model predictions to find the best possible combination of the two models for maximum prediction power. Each data point represents the correlation between the observed and combined prediction from both models. The combined prediction is calculated by combining the prediction output from both models in different percentages (the x-axis shows the weight percentage of the linear model). (**F**) Combined final model. Correlation plot between the observed and the combined prediction from both models. The prediction output is a combination with 50% contribution from both models.

We again used 90% of genome-wide screening data to train the linear model and used the same data to extract an average seed phenotype. These two factors where independently used to predict the remaining 10% of data for both infection and cell count phenotypes. Figure 4A shows that the linear regression model was able to predict the cell count with up to 55% accuracy whereas it has again no predictive power on the infection phenotype (Figure 4D). Contrary to this, seed models could predict both cell count and infection phenotypes up to 55% and 54%, respectively (Figure 4B and E). One caveat of using seed models for prediction purposes is the restricted availability of seed information in the training data. Not all possible seed sequences are represented in genome-wide screens, which is also shown by the vertical line at position *zero* in Figure 4B and E, reflecting test oligos for which no prediction could be made.

We then combined these two models in an effort to enhance the overall prediction power for the cell-count. This was done by averaging the prediction output from both models in a weighted manner (Figure 4C). We observed that the final model, at 50% contribution each from the nucleotide composition model and the seed effect model (Figure 4F), was able to increase the accuracy of the cell count prediction from 55% (single models) to 65% (combined model). This superior combined model relies on average seed phenotype information, which may not always be available. In such cases, where a lack of seed representation in the library prevents a combined analysis, the linear nucleotide composition model can still be used – it is applicable to any oligo, and furthermore requires much less data to train.

### Growth-restricting effects of siRNA transfections may be due to competition with endogenous processes

It has been shown in previous studies that transfected siRNAs can reduce the viability of cells due to a variety of unintended effects, some of which are related to innate immune responses and/or to possible saturation of the endogenous RNAi machinery (27–29,31).

We experimentally investigated both these above scenarios. First, we conducted a RT-qPCR experiment to detect any upregulation of innate immune marker genes in response to transfection with customized siRNAs. This was done at two time points (12 h and 24 h post transfection) (Supplementary Figure S1). The RT-qPCR experiments showed no significant immune response for any customized siRNA at either exposure interval. Secondly, we set up a competition experiment, by transfecting cells with a standard siRNA having a known on-target effect, combined with various test oligos of customized sequence. By first establishing the dose-response curve for the known, well characterized siRNA alone, we were then able to detect any competitive influences of other co-transfected oligos, via horizontal shifts in that dose-response curve. We tested three kinds of potential competitor oligos, namely those predicted by our algorithm to be fitness-reducing or non-fitness-reducing, plus randomly sampled background controls. All competitor oligos were applied as custom-designed, arbitrary sequences, with no intended on-target.

**Figure 5.**
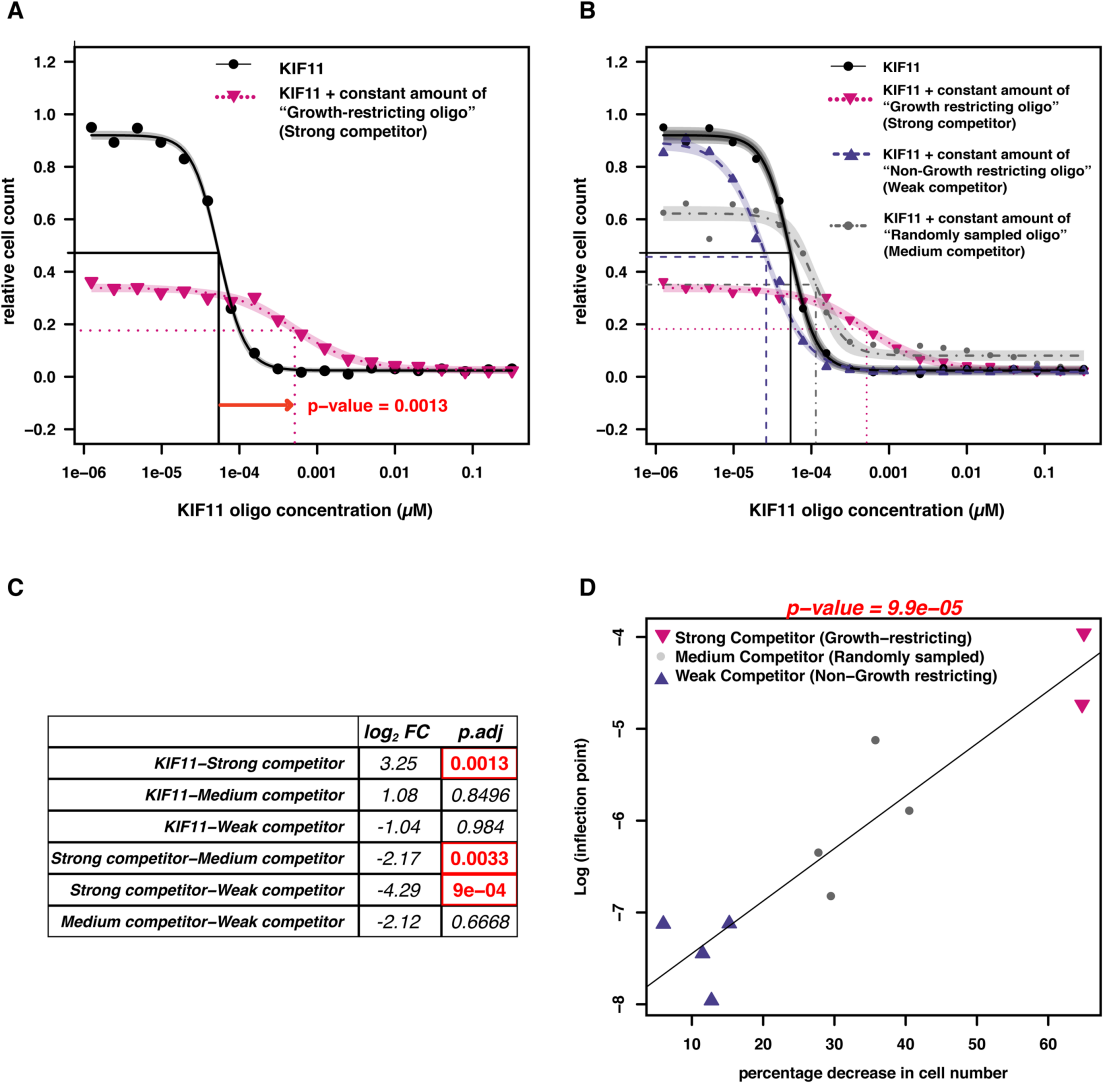
Co-transfected siRNAs compete with one another in a predictable manner. (A) A titration experiment was set up to establish a dose-response curve for a standard siRNA targeting the KIF11 transcript, which has a strong on-target effect (cell death) and is thus used as a transfection control in many screens. (**A, B**) The black curve from (A) was repeated with various oligos co-transfected at a constant amount, and the horizontal shift of the dose-response curve was measured. (**C**) Significance of shifts in concentration response curves upon co-transfecting various classes of siRNAs (in each pairing, the left oligo was titrated, and the right was given at a constant amount; FC = fold change, dividing the right oligo's inflection point by the left oligo's inflection point). (**D**) Correlation plot between shifts in concentration response curves of siRNAs targeting KIF11 upon co-transfection with customized siRNAs, and decrease in cell number upon transfection with these customised oligos alone.

This experimental competition setup (Figure 5A, B) indeed revealed a strong competitive interaction between the various customized siRNAs and a known on-target siRNA (the latter is designed to target the KIF11 transcript; KIF11 is essential for cell division and its knockdown leads to strong cell death). Remarkably, co-transfected siRNAs can shift the dose-response curve of the KIF11 oligo towards *higher* viability (Figure 5A). This is particularly striking, as the competitor siRNAs themselves had been selected due to their capability to reduce cell numbers (i.e. cellular fitness/proliferation). In our view, this counter-intuitive outcome can be explained only by competition, presumably for some rate-limiting component of the RNAi machinery. The shift observed in Figure 5A is statistically significant, and shows that a tenfold higher concentration of KIF11 is required in order to inhibit cell growth in presence of a growth-restricting siRNA. This competition varied in a predictable manner with the various sequences of co-transfected siRNAs (Figure 5B) as seen by the different significant shifts in the concentration response curve of siRNA targeting KIF11 (Figure 5B, C). The degree to which a customized siRNA was competing was directly proportional to its growth-restricting effect when applied alone, whereby the most effective growth-restricting siRNAs were also the strongest KIF11 competitors (Figure 5D).

The above observations lead us to hypothesize that the different classes of competitor siRNAs have different binding preferences for one or several components of the RNAi machinery, and hence can compete more or less efficiently with other oligonucleotides binding to these components. This would allow them to saturate the machinery to different degrees as shown by the distinct shifts in concentration response curves elicited by them.

If correct, our hypothesis would predict that these competitive effects between siRNAs should happen somewhere along the miRNA processing pathway, at or upstream of the RISC complex. We therefore tested the involvement of Argonaute, the central RISC component, by lowering active Argonaute concentration through AGO2 siRNA knockdowns (Supplementary Figure S2). First, we observed that AGO2 knockdowns reduced cellular fitness/proliferation already by themselves, without any further oligos added (cell counts were reduced by roughly 20%, relative to GFP control knockdowns). This is consistent with a central role of AGO2, and the fact that AGO2 is essential, already for early embryonic development (41). Next, we tested the effect of AGO2 reduction on the function of a given on-target siRNA, using the strong KIF11 siRNA cell death phenotype, as above. Remarkably, AGO2 knockdown leads to a strong rescue of the KIF11 phenotype, rescuing cell numbers from <200 to >900 (Supplementary Figure S2). This indicates that the RISC complex is indeed functionally required for the action of siRNAs, at least with respect to their on-target action. In a last step, we then tested the effects of AGO2 knockdowns on several dozen of our custom-designed competitor siRNAs (none of which have any intended on-target). In the knockdown, the difference between strong and weak competitors, which normally amounts to a cell number difference of more than 50%, is strongly reduced, with a remaining difference of <20%. Importantly, relative to a control knockdown using siGFP, an AGO2 knockdown actually leads to *enhanced* cell numbers in the case of strong competitor oligos, amounting to a rescue of ∼45% (Supplementary Figure S2, $P = 5 \cdot {10^{ - 8}}$). Together, these observations are consistent with a scenario in which the competition takes place somewhere along the miRNA processing pathway, a mechanism that presumably can affect endogenous miRNAs similarly.

It has been observed in previous studies that changes in the expression of endogenous miRNAs upon transfection with certain siRNAs generally lead to noticeable changes at genomic and proteomic levels (29–31). These might then give rise to the sequence-dependent and predictable cell-count phenotypes that we observe here, ranging from strongly reduced cellular fitness/proliferation to hardly any effect.

## DISCUSSION

A full understanding of the unintended side-effects of siRNA transfections is particularly important in the context of therapeutic applications, or in quantitative genetics experiments (e.g. double knockdowns in epistasis settings). Those siRNA sequences that are particularly problematic for cellular fitness / proliferation, as predicted by our model, could be avoided in such settings – or used in lower dosage to reduce the observed effects. Our co-transfection experiments suggest that competition between co-transfected siRNAs may reduce the efficacy of any individual siRNA in a given experiment, therefore the results of double-knockdown epistasis perturbations need to be interpreted with caution.

How exactly the reduction in cellular fitness/proliferation comes about mechanistically may be difficult to unravel: first, the effect—albeit universally observed in all libraries and cell lines that we tested—is relatively mild. After 72 hours of siRNA transfection, the strongest observed reduction in cell numbers is only ∼48%, with the overall strength of the effect depending on the type of library and the transfection conditions. Second, it is conceivable that the observed phenotype is the result of multiple mechanistic insults, brought about by several small changes in endogenous miRNA efficiency followed by pleiotropic downstream effects. Third, the effect can be easily masked in any given oligo of interest, by the (stronger) impact of seed-directed, hybridization-dependent off-target binding. Nevertheless, systematic measurements of endogenous, target-bound miRNAs in the absence or presence of strong ‘fitness-reducing’ competitors may reveal sets of affected miRNAs which can be followed up experimentally in the future. It should be noted that the other, independent phenotype that was studied here (the infection phenotype) does not show any discernible nucleotide-by-nucleotide effects (Figure 2B). This might indicate that this phenotype is under the control of a smaller number of genes; pathogens might also have evolved to be robust against small perturbations in the quantitative makeup of the host cell.

What does our observation imply for high-throughput screening? At least for the human KIF11 gene, which we tested here in detail, our dose-response curves suggest that the oligo concentrations that are routinely used in high-throughput screens are fairly high—certainly well into the fully saturated regime. This may be problematic since any excess amount of oligo is free to interfere with cellular fitness/proliferation, but no longer increases the desired knock-down effect on the target gene. There are several strategies conceivable to improve the situation. One strategy is to pool several distinct siRNAs for each intended target gene, either explicitly or by slicing up longer precursor RNAs (e.g. esiRNAs/siPools) (42,43). This lowers the concentration of each individual molecular species, and may average out the competitive effects to some degree.

Alternatively, staying with individual siRNA species in order to have better-defined perturbations, one might need to lower the overall concentrations at which these oligos are used in screens; this would require carefully titrating the intended assays/readouts using applicable positive controls (so as to avoid going too low). Perhaps more promising, however, would be a two-fold approach: first, to avoid problematic sequences already at the library design stage as they can now be fairly confidently predicted *in silico*, and second, to move away from uniform abundance distributions of siRNAs in libraries. After all, in many cases the expected relative concentrations of the intended target mRNAs in the cell are well known or can be measured easily. Then, adjusting the stoichiometries of the siRNA molecules in the libraries accordingly, would give a better overall fit between the perturbing reagents and their targets. This would allow the titration of the whole library—en bloc— toward an optimal transfection condition for any given screen, balancing knockdown efficiency versus non-specific side effects.

## Supplementary Material

Supplementary Data
