# Supplementary material for: Growth-restricting effects of siRNA transfections: a largely deterministic combination of off-target binding and hybridization-independent competition
Source: Nucleic Acids Res. 2018 Sep 12;46(18):9309–20. doi: 10.1093/nar/gky798 (PMC6182159; doi:10.1093/nar/gky798)
Supplement: Supplementary Data [file gky798_supplemental_files.zip › supplementary_information.pdf]

Supplementary Information

Growth-restricting effects of siRNA transfections: a largely deterministic combination of off-target binding and hybridization-independent competition

Neha Daga[1,2], Simone Eicher[3,+], Abhilash Kannan[1,+], Alain Casanova[3], Shyan H. Low[3], Saskia Kreibich[4], Daniel Andritschke[4], Mario Emmenlauer[3], Jeremy L. Jenkins[5], Wolf-Dietrich Hardt[4], Urs F. Greber[1], Christoph Dehio[3], and Christian von Mering[1,2,*]

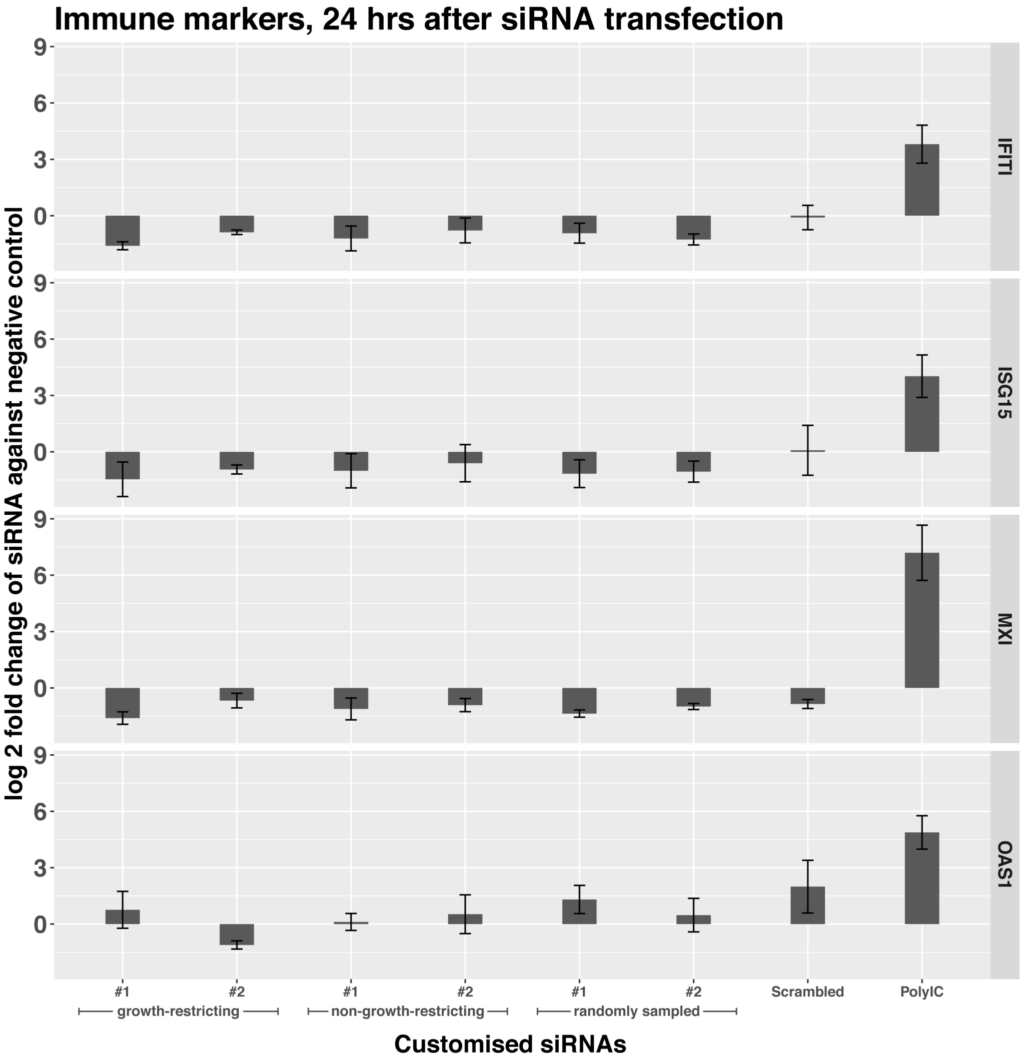

Supplementary Figure S1:
Expression levels of immune marker genes upon transfection with customized siRNAs.
To measure the levels of Human MX1, IFIT1, ISG15 and OSA1, HeLa CCL-2 cells were reverse-transfected with 20 nM siRNAs using 0.1% (v/v) Lipofectamine RNAiMAX (Invitrogen), according to the manufacturer's instructions. Transfection reactions were performed in serum-free OptiMEM (Gibco). Total RNA from the siRNA transfected cells was extracted using mirVana RNA isolation kit (Ambion). Following on-column DNase digestion (Qiagen), 300 ng of total RNA was reverse-transcribed using the 'Transcriptor' First Strand cDNA Synthesis Kit (Roche) with anchored-oligo(dT)$_{18}$ primers. RT-qPCR was performed using Power SYBR Green Master mix (Applied Biosystems). Reactions were analysed by an ABI 7000 real-time PCR machine using the following cycle conditions: 50°C for 10 minutes, 95°C for 10 minutes, followed by 40 cycles at 95°C for 15 seconds and 60°C for 1 minute. Relative mRNA levels ($2^{-\Delta Ct}$) were determined by comparing the PCR quantification cycle (Cq, determined with the Software SDS 2.2.1) and normalized against the reference gene expression TATA-Box Binding Protein (TBP), elongation factor 1 alpha (EEF1A1) and transferrin receptor protein 1 (TFRC). The differences in their Ct cycles were calculated (ΔΔCt). Column labels (x-axis): designations of the transfected siRNA oligos – these are custom-designed oligos not intended to have any on-target in the human genome (same oligos as used in Fig. 5; see Supplementary Table S2 for sequences).

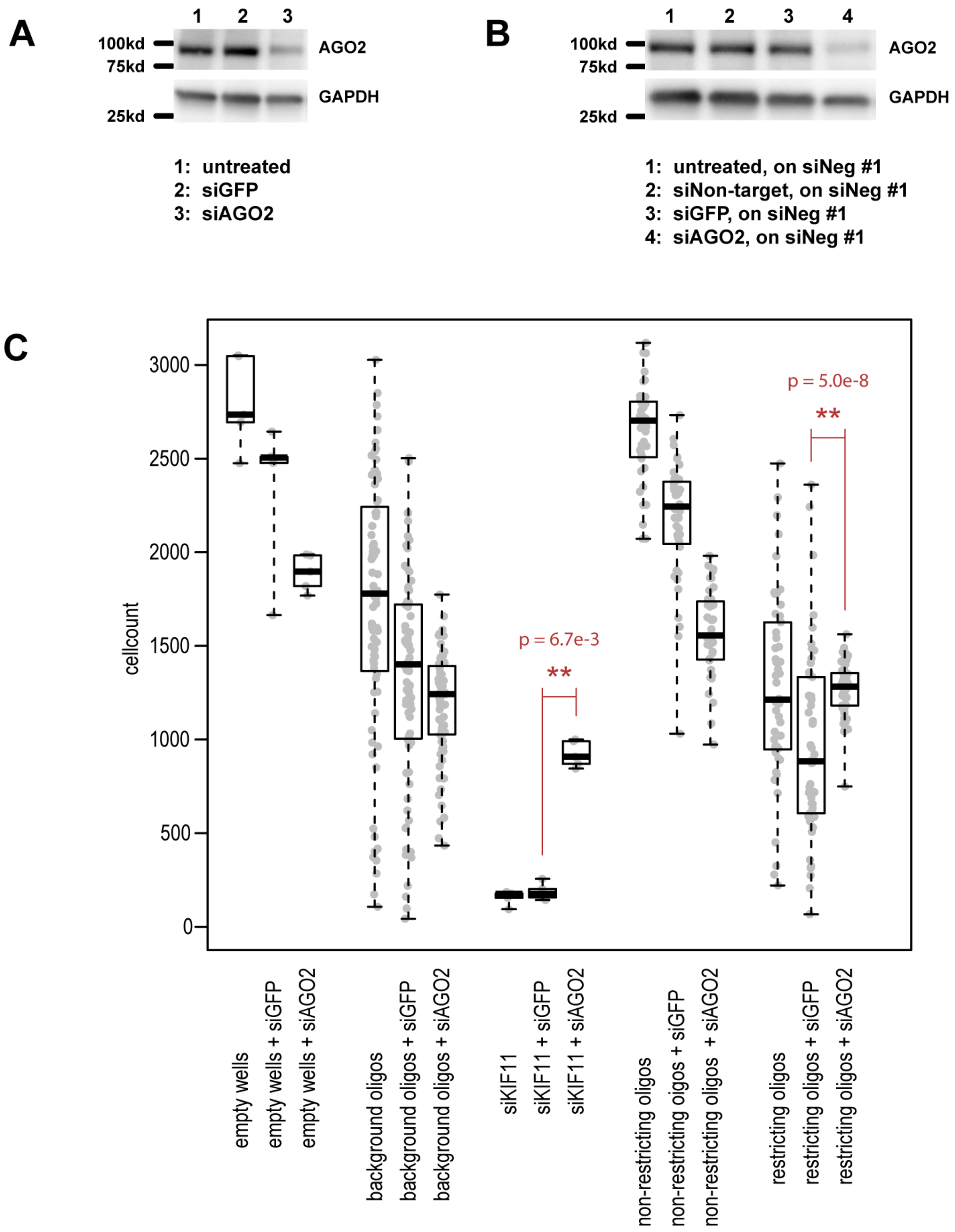

Supplementary Figure S2:

AGO2 knockdowns can partially rescue siRNA-mediated growth restriction.

A) Western Blot of HeLa whole-cell extracts 120 hours after knocking down AGO2 by means of siRNA transfection; the protein levels of AGO2 are seen strongly reduced. This is not observed in a control experiment where an siGFP oligo was used instead (the cells do not carry any GFP construct).

B) experiment similar to A), but after 48 h of AGO2 knockdown, cells were transferred for a second transfection onto a negative control siRNA for another 72 h. The non-targeting control siRNA does not interfere with the AGO2 knockdown.

C) Cells exposed to 48 h of AGO2 knockdown were transferred onto a 384-well plate pre-seeded with various siRNAs of interest. 72 h later, cell numbers were read out for each well. Relative to empty wells, the addition of all oligos reduced cell-numbers, even in the case of siGFP, which had no on-target in the cells. The strongest reduction in cell-number is achieved by siRNAs targeting the KIF11 gene (known on-target mechanism). This was strongly reversed by the AGO2 knockdowns; similarly, the growth-restricting effects of custom-designed siRNA oligos without intended on-target was also reversed by the AGO2 knockdown (relative to the GFP control knockdown). The siRNAs tested on the 384-well plate were the same as in Figure 3 of the main manuscript; their sequences are available in the Supplementary Data file “customized_oligos_all_cells.xlsx”. The AGO2 knockdown was achieved with a Dharmacon “SMARTpool” reagent – the four oligos in the pool are not predicted by our algorithm to be unusually growth-restricting (average predicted z-score is -0.02).

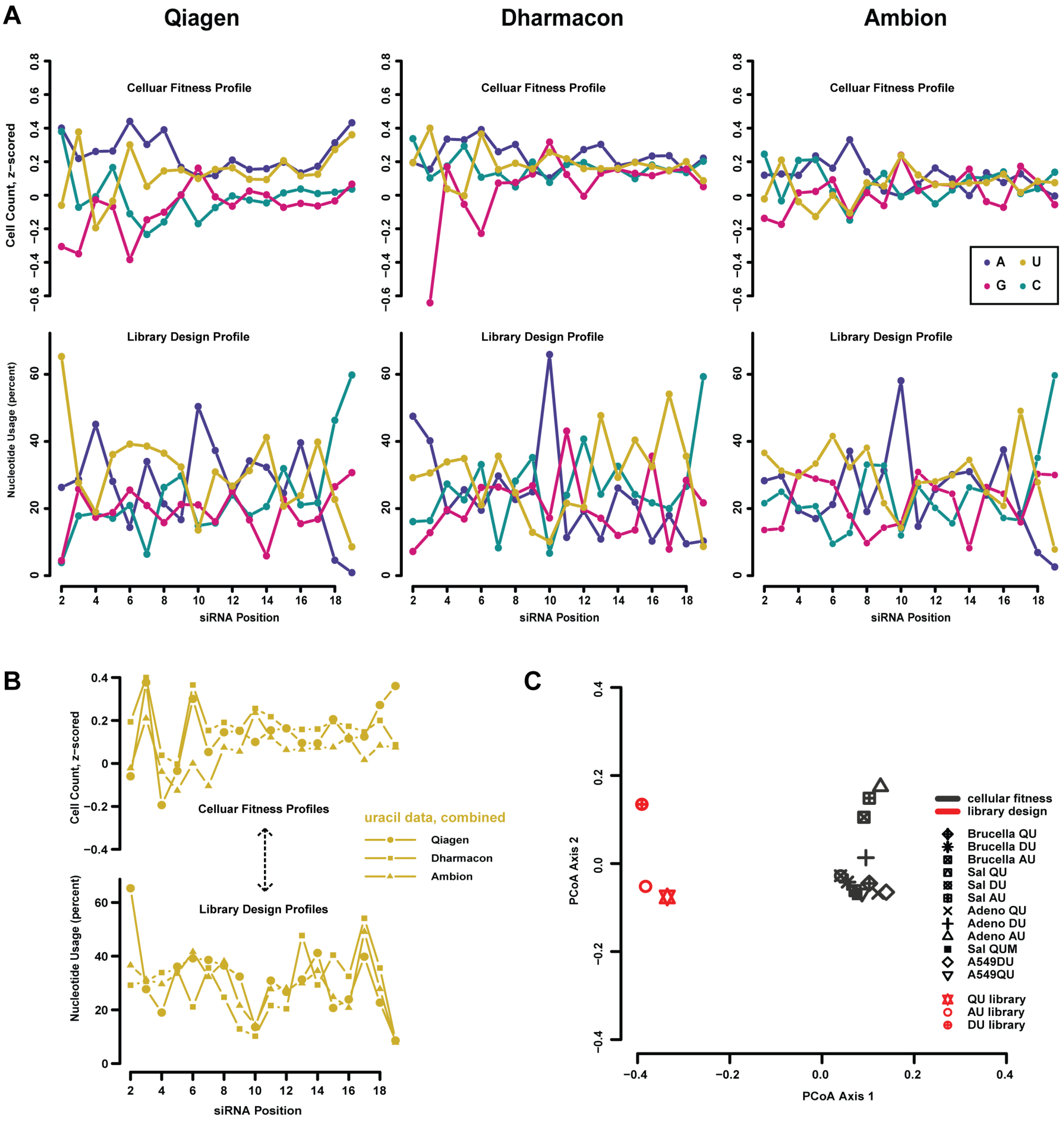

Supplementary Figure S3:

Comparing position-specific library design biases with position-specific phenotypic consequences.

A) For each of the three commercial siRNA libraries considered in this study, the top panel shows the average cell count phenotype of all oligos that happen to have a given nucleotide at a given position. These have been computed and plotted in exactly the same way as show in Figure 2A of the main manuscript. In contrast, the bottom panels show the usage frequencies of the various nucleotides at the various positions as designed by the library providers. These profiles reflect choices made to increase effectiveness and specificity of the oligos. The outermost 5' position (position #1) shows a very strong design bias in all libraries (mostly "U") and was thus not considered in our study. For the Qiagen library, the siRNA sequences are shortened at the 3'-end, so that all profiles have the same length here (from position #2 to position #19).

B) same data as in A, but combining all libraries into one plot and showing only the data for "U" (uracil), to allow for a direct, exemplary comparison.

C) here, all four nucleotide profiles for a given library have been concatenated and their values converted to ranks. Then, they are compared in a PCoA analysis, revealing that the biases are distinct from the phenotypes.

primer name	primer sequence
MX1-forward	5'-TTC AGC ACC TGA TGG CCT ATC-3'
MX1-reverse	5'-TGG ATG ATC AAA GGG ATG TGG-3'
IFIT1-forward	5'-GCG CTG GGT ATG CGA TCT C-3'
IFIT1-reverse	5'-CAG CCT GCC TTA GGG GAA G-3'
OAS1-forward	5'-AGC TTC GTA CTG AGT TCG CTC-3'
OAS1-reverse	5'-CCA GTC AAC TGA CCC AGG G-3'
ISG15-forward	5'-GAG AGG CAG CGA ACT CAT CT-3'
ISG15-reverse	5'-AGC TCT GAC ACC GAC ATG G-3'

Supplementary Table S1:
Nucleotide sequences of all qPCR primers used for mRNA quantification in Supplementary Figure S1 (i.e., measuring expression levels of immune marker genes upon transfection with customized siRNAs).

siRNA oligo	catalogue name	sequence (guide str., 5'-3')	catalogue ID	provider
KIF11	Hs_KIF11_4 FlexiTube siRNA	UAUACUGAGAAAGCCAUCUAG	SI00064855	QIAGEN
non-growth-restr. 1	HP Custom siRNA	UAUAUAAUGAUUAUAAUUUTT	custom	QIAGEN
non-growth-restr. 2	HP Custom siRNA	UCUUAAAUAUCUAAAGCGUCT	custom	QIAGEN
randomly sampled 1	HP Custom siRNA	UAGAUUAUUAAUCGAAUCCAG	custom	QIAGEN
randomly sampled 2	HP Custom siRNA	UUCCUUAACGAUAACACCGCG	custom	QIAGEN
growth-restricting 1	HP Custom siRNA	AGUGUGGGUCACCUUCCGCAG	custom	QIAGEN
growth-restricting 2	HP Custom siRNA	UUCUAGGCGACGCUGUAGCTG	custom	QIAGEN
siAGO2	Ago2 (EIF2C2) SMARTpool siRNA	UUCAGAUGGACUUCCGUGC<br>UAAUACAUCUUUGUCCUGC<br>UAUUUAUCACCACAGACCC<br>UGACAUUGGGUUCUCAUAC	D-004639-01<br>D-004639-02<br>D-004639-03<br>D-004639-04	Dharmacon
siGFP	GFP Duplex III	GAACUUCAGGGUCAGCUUGCC	P-002048-03	Dharmacon
siNon-target	ON-TARGETplus Non-targeting 1	UUAGUCGACAUGUAAACCA	D-001810-01	Dharmacon
siNeg #1	Negative Control No.1 siRNA	not disclosed by provider	4360844	Ambion

Supplementary Table S2:
Nucleotide sequences of all siRNA oligos used for Figure 5 or for Supplementary Figure S2.
Note that the siRNA oligo named “growth-restricting 1” resulted in a cell count phenotype that was too severe to allow the determination of an inflection point in the KIF11 dose-response curve when used in co-transfections. It was hence not used for Figure 5, and it is listed here merely for completeness.
